# Inferring HIV Escape Rates from Multi-Locus Genotype Data

**DOI:** 10.3389/fimmu.2013.00252

**Published:** 2013-09-03

**Authors:** Taylor A. Kessinger, Alan S. Perelson, Richard A. Neher

**Affiliations:** ^1^Evolutionary Dynamics and Biophysics, Max Planck Institute for Developmental Biology, Tübingen, Germany; ^2^Theoretical Biology and Biophysics, Los Alamos National Laboratory, Los Alamos, NM, USA

**Keywords:** HIV, CTL escape, cytotoxic T-lymphocytes, HIV evolution, viral dynamics, selection coefficient

## Abstract

Cytotoxic T-lymphocytes (CTLs) recognize viral protein fragments displayed by major histocompatibility complex molecules on the surface of virally infected cells and generate an anti-viral response that can kill the infected cells. Virus variants whose protein fragments are not efficiently presented on infected cells or whose fragments are presented but not recognized by CTLs therefore have a competitive advantage and spread rapidly through the population. We present a method that allows a more robust estimation of these escape rates from serially sampled sequence data. The proposed method accounts for competition between multiple escapes by explicitly modeling the accumulation of escape mutations and the stochastic effects of rare multiple mutants. Applying our method to serially sampled HIV sequence data, we estimate rates of HIV escape that are substantially larger than those previously reported. The method can be extended to complex escapes that require compensatory mutations. We expect our method to be applicable in other contexts such as cancer evolution where time series data is also available.

## Introduction

During the first few months of HIV infection, the HIV genome typically undergoes a series of rapid amino acid substitutions that reduce immune pressure by cytotoxic T-lymphocytes (CTLs); this process is referred to as CTL escape (1). The substitutions arise by random mutation and spread through the viral population by impairing either the presentation of viral epitopes on the cell surface or the recognition of the viral epitope by T-cell receptors. Avoiding recognition is an obvious benefit to the mutant virus, but escape mutations can interfere with processes necessary for virus replication and infection and thereby reduce the virus’ intrinsic fitness (2–5). The rate at which escape variants displace the founder sequences depends on both “avoided killing” and the fitness cost. To quantify the role of individual CTL clones in controlling the viral population and the fitness costs associated with escape mutations, one would like to infer the escape rate associated with the individual mutations from serially sampled sequence data (4, 6).

**Figure 1:**
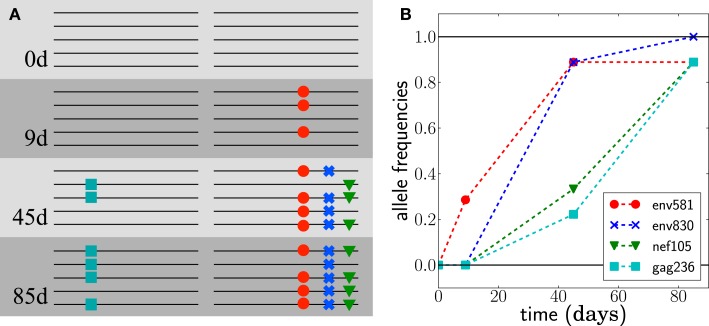
**Escape from T-cell mediated immunity**. The virus population in patient CH58 quickly acquires four substitutions. **(A)** shows a sketch of genotypes at the first 4 escape mutations, observed at different times; see (7, 8) for the actual data. **(B)** shows the frequencies of the mutations in samples of size 7 at day 9 and size 9 at days 45 and 85.

With a single escape mutation and dense, deeply sampled data, the escape rate can simply be estimated by fitting a logistic curve to the time course of the mutation’s frequency (4, 6). The logistic curve has two parameters: the growth or escape rate and the frequency at the initial time point. In many cases, however, the data obtained from infected patients are scarce, and estimating two parameters reliably from the data is not possible since one needs at least two time points at which the mutation is at intermediate frequency between 0 and 1 (4). Figure 1 shows an example of such time series sequence data from CTL escape during early HIV infection. Time points are far apart and the sampling depth is low. Furthermore, it is not the case that only a single escape mutation is observed; rather, several mutations rapidly emerge in different places in the viral genome (7, 8). Multiple escapes imply immune pressure on many epitopes. Since the viral population and its mutation rate are large (9, 10), these different escape mutations will arise almost simultaneously. Initially, these escape mutations exist in the population as single mutant genomes until they are combined into multiple mutants by recurrent mutation or recombination (11, 12). The competition between viral variants affects the trajectories of individual escape mutations, so estimating their intrinsic growth rate by logistic fitting is not accurate. This competition is known as “clonal interference” in population genetics. The degree of competition between genotypes depends on the population size, the mutation rate, and the recombination rate in HIV populations. The latter-most is rather low (13, 14), and two strongly selected mutations in a large population are more likely to be combined by additional *de novo* mutation than recombination with another rare single mutation.

Here, we develop a strategy for inference that allows one to obtain robust escape rate estimates from the scarce data typical of studies of CTL escape. The inference is based on explicit modeling of the process of mutation accumulation in the founder sequence. Thereby, we exploit constraints imposed by the underlying dynamics of mutation and selection in the high dimensional space of possible genotypes.

Despite the large number of possible genomes that can be formed from different combinations of escape mutations, we typically observe one or two dominant genotypes at a time – at least during the first few months of the infection. Furthermore, these genotypes dominate only transiently and are quickly displaced by genotypes with an even greater number of escape mutations; see Figure 1. These observations agree with results from ref. (15), where a model of acute HIV infection was used to show that strongly selected escape mutations fix sequentially. Note that we don’t assume a particular sequence of dominant genotypes *a priori*. Instead, we observe a sequence of dominant genotypes and try to infer the evolutionary scenario that most likely gave rise to this sequence of genotypes. While we model only these genotypes, many minor variants certainly exist. But only those dominant variants that are likely to give rise to the future populations need to be modeled accurately. Later in infection, the viral population is very diverse and cannot be analyzed using our method.

Given a data set from early infection, it is typically straightforward to define a series of dominant genotypes that likely have arisen through step-wise accumulation of mutations. Note that most likely all escape mutations constantly arise in different combinations, but typically only one combination rises quickly enough to dominate the population. This dominant genotype is then in most cases the source for the next dominant genotype. Later in infection, however, recombination is sufficiently frequent that no dominant genotype exists and mutations can spread simultaneously.

In Ganusov et al. (11), a framework for multi-locus modeling of CTL escape is presented. Building on this framework, we explicitly model the transition from one dominant genotype to another, which is a good approximation of the dynamics for rapid CTL escape in acute infection. The restriction to dominant genotypes captures the interference between escapes at different epitopes while avoiding the need to solve the full multi-locus problem.

We will first define a model of the dynamics of escape mutations. This model serves a twofold purpose: it defines the parameters we would like to estimate from the data and provides us with a computational tool to investigate how the accuracy of the inference depends on sampling depth and frequency, as well as how sensitively it depends on the values of parameters such as mutation rates or the population size. We reanalyze existing CTL escape data and find that accounting for multi-locus effects in a finite population results in higher estimates of the escape rates.

## Results

### Model

In the majority of sexually transmitted HIV infections, a single “transmitted/founder” virus initiates the new infection resulting in an initially homogeneous viral population (8, 16). However, as HIV replicates in its new host, mutations accumulate. Mutations within or in proximity to CTL epitopes can reduce immune pressure by facilitating the avoidance of CTL recognition. While one often observes several escape mutations within a single epitope (17, 18), we do not differentiate between different mutations within the same epitope and model *L* epitopes that can be either be mutant or wild-type. Assuming that the escape at multiple epitopes has additive effects, ε*_j_*, the growth rate (birth rate minus death rate) of a genotype is given by

(1) $$F\left(g,t\right)=F_{0}\left(t\right)+\sum_{i}\varepsilon_{i}s_{i}$$

where *g* = {*s*_1_, …, *s_L_*} specifies the genotype. Here, *s_i_* = 0 corresponds to a wild-type epitope at locus *i*, whereas *s_i_* = 1 signifies escape at that epitope. *F*_0_(*t*) accounts for a genotype independent modulation of the growth rate. The latter could, for example, be due to variable numbers of target cells (19, 20). *F*_0_(*t*) controls the total population size, while the differences between genotypes are accounted for by $\sum_{i}\varepsilon_{i}s_{i}$ and result in differential amplification of some genotypes over others. The ε*_i_* are the escape rates that we would like to estimate from the data and should be interpreted as the net effect of avoided killing and the possible fitness costs associated with the mutation; see e.g., Ganusov et al. (11). The fitness costs are modulated by the overall growth rate of the viral population and could therefore be slightly time dependent. We neglect this complication. Within our model, mutations arise at a rate μ per base per generation. This rate can be epitope dependent. Motivated by the frequent template switching of HIV reverse transcriptase (21), our general model of the HIV population includes recombination, which is assumed to occur with rate *r*. In the event of recombination, all *L* epitopes are reassorted, but an explicit genetic map could be implemented as well.

We implemented our model as a computer simulation in Python using the population genetic library FFPopSim (22). The simulation stores the population *n*(*g, t*) of each of the 2*^L^* possible genotypes. In each generation, the expected changes of the *n*(*g, t*) due to mutation, selection, and recombination are calculated. The population of the next generation is then sampled from the expected genotype frequencies γ(*g*, *t*) = *n*(*g*, *t*)/*N*. The size of the population, *N*, can be set at will each generation. In this way, up to 15 epitopes can be simulated for 1000 generations within seconds to minutes.

**Figure 2:**
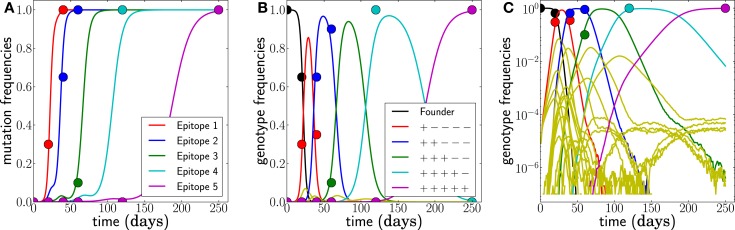
**Example of simulated escape mutations spreading through the population**. **(A)** Even though all epitopes are targeted from *t* = 0, escape mutations spread sequentially. The mutation frequency in a sample of size 20 at different time points is indicated by colored dots. **(B)** The rising mutation frequencies are associated with the rise and fall of multi-locus genotypes. The founder virus is first replaced by a dominant single mutant, which itself is replaced by a double mutant and so forth. Note, however, that the virus population explores many combinations of mutations but that these minor variants never reach appreciable frequency. This is best seen in **(C)**, where all 32 genotype frequencies are shown on a logarithmic scale. These rare variants are rarely sampled, and their noisy dynamics suggests that little information can be gained from them. Here, *N* = 10^7^, μ = 10^−5^, and *r* = 0, and escape rates are ε*_j_* = 0.5, 0.4, 0.25, 0.15, 0.08 per day.

A typical realization of the population dynamics is shown in Figure 2, where we have assumed a generation time of 1 day. As expected, the population is dominated by one genotype at a time. Furthermore, the mutations accumulate in decreasing order of escape rate, and the new dominant genotype arises from the previous by incorporation of the mutation with the largest escape rate available. There are, however, many minority genotypes which are rarely observed. Figure 2C shows the frequencies on a logarithmic scale, where the minor variants are visible. We use these simulations to test the accuracy and robustness of the inference procedure developed below.

Of the many possible genotypes that are present at any moment, only a small fraction is likely to be observed in a small sample and to be relevant in the future. Simulations and data suggest that the dominant genotypes accumulate mutations one by one – this greatly simplifies the task of estimating escape rates from the data. Instead of considering the dynamics of all possible genotypes (2*^L^*), we will restrict the inference to a chain of genotypes, each containing one additional mutation compared to its predecessor.

The best estimate for the HIV generation time is *d* = 2 days (23), while estimates of escape rates are typically given in units of inverse days rather than generations. For simplicity, we simulate our model assuming one generation per day and state all rates in units of 1/day. Our results are insensitive to the choice of the generation time. Doubling the generation time has similar effects to dividing the population size by 2, as this keeps the strength of genetic drift constant.

### Inferring the escape rates

Suppose we have obtained sequence samples of size *n_i_* at different time points *t_i_* and each of these samples consists of different genotypes *g* present in *k*(*g, t_i_*) copies. If the actual frequencies of those genotypes at different times are γ(*g, t_i_*), the probability of obtaining the sample at *t_i_* is given by the multinomial distribution

(2) $$P\;\left(\mathrm{sample}\right)=\frac{n_{i}!}{\prod_{g}k\;(g,t_{i})!}\prod_{g}\gamma \;{\left(g,t_{i}\right)}^{k(g,t_{i})}$$

If the underlying dynamics was deterministic, the frequencies γ(*g, t*) would be unique functions of the model parameters we want to estimate. In that case we could use Bayes’ theorem, choose suitable priors, and determine the posterior distribution of the parameter values. However, both the model and the actual viral dynamics are stochastic, and “replaying” the history would result in different trajectories. Furthermore, most of the 2*^L^* possible genotypes remain unobserved. This leaves us with the choice of either some type of approximate Bayesian computation that compares repeated simulations of the model with appropriate summary statistics (24) or a reduced description of only the observed genotypes, with the stochasticity captured by nuisance parameters (25).

We opt for the latter and model only those genotypes that dominate the population. We label these genotypes by the number of escape mutations they carry, e.g., *g*_1_ carries the first escape mutations, *g*_2_ the first and the second, and so forth. The frequency of a genotype is affected by stochastic forces only while it is very rare. If the genotype is favored, it will rapidly rise to high frequency, and the stochastic effects will no longer be relevant. It is therefore convenient to summarize the stochastic behavior by the time, τ, at which its frequency crosses the threshold to essentially deterministic dynamics. Since the dynamics is deterministic after this “seed time,” all the (unobserved) stochasticity can be accounted for by an appropriate choice of the seed time (26, 27). For each of the dominant escape variants, *g_j_*, with *j* = 1 to *j* = *L* escaped epitopes, we define a seed time τ*_j_* to accommodate the stochastic aspects of the escape dynamics.

**Figure 3:**
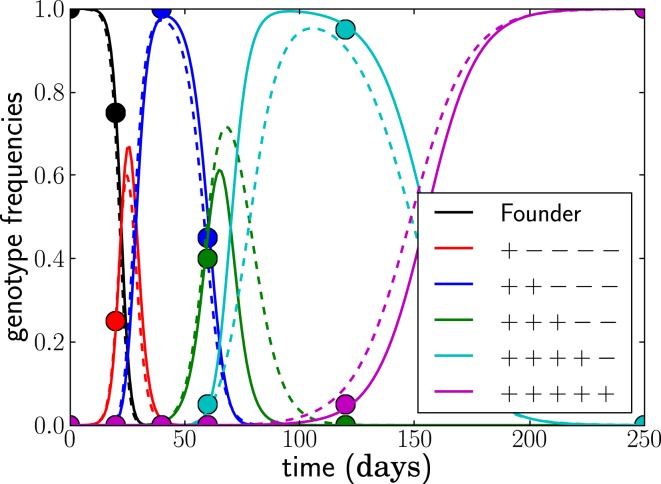
**The deterministic model parameterized by seed times τ*_j_* for the *L* dominant genotypes and the escape rates of epitopes ε*_i_* (solid lines) captures the dynamics of the stochastic model accurately (dashed lines)**. The trajectories (and seed times) vary from run to run. In this run, *N* = 10^7^, μ = 10^−5^, and *r* = 0 and the escape rates are ε*_j_* = 0.5, 0.4, 0.25, 0.15, 0.08 per day.

After crossing the deterministic threshold, the population frequencies of the dominant genotypes evolve according to

(3) $${\dot{\gamma }}_{j}\left(t\right)=F\left(g_{j},t\right)\gamma_{j}\left(t\right)+\mu \left[\gamma_{j-1}\left(t\right)-\gamma_{j}\left(t\right)\right]$$

if *t* > τ*_j_*. Conversely, γ*_j_*(*t*) = 0 for *t* < τ*_j_*. The growth rate *F*(*g_j_, t*) of genotype *j* is the sum of the escape rates ε*_k_* of the epitopes *k* = 1, …, *j* and the density regulating part *F*_0_(*t*); compare to equation (1). The escape rates are what we would like to estimate. The seed time, τ*_j_*, corresponds to the time at which a genotype with all escape mutations up to mutation *j* first establishes^1^. At the seed time, we initialize the genotype frequency at γ*_j_*(τ*_j_*) = *N*^−1^. If seed times are chosen appropriately, this model provides a very accurate description of the frequency dynamics of the dominant genotypes in the full stochastic model; see Figure 3.

At face value, the deterministic model has two parameters per epitope – one escape rate and one seed time. The seed times, however, are quite strongly constrained by basic facts of the evolutionary dynamics. The genotype *g_j_* carrying mutations *i* = 1, …, *j* arises with rate μ*N*(*t*)γ*_j_*_ − 1_(*t*) from the genotype *g_j_*_ − 1_ carrying only *j* − 1 mutations. This means it is unlikely that genotype *j* arises early while γ*_j_*_ − 1_(*t*) is still very small. However, once the previous genotype *j* − 1 is common, genotype *j* is produced frequently. The distribution of the time at which the first copy of genotype *j* arises is given by the product of the rate of production and the probability that it has not yet been produced. The latter is the negative exponential of the integral of the production rate up to this point. Hence, the distribution of the seed time τ*_j_*, given the trajectory of the previous genotype γ*_j_*_ − 1_, is given by

(4) $$Q\left(\tau_{j}|\gamma_{j-1}\left(t\right)\right)\approx \mu N\left(\tau_{j}\right)\gamma_{j-1}\left(\tau_{j}\right)e^{-\mu \int_{0}^{\tau_{j}}N\left(t\right)\gamma_{j-1}\left(t\right)\;\mathrm{dt}}.$$

Since the γ*_j_*(*t*) are uniquely specified by {τ*_k_*, ε*_k_*}*_k_* _= 1, …_, *_L_*, we can write the posterior probability of the parameters as

(5) $$P\left(\left\{\varepsilon_{j},\tau_{j}\right\}\right)\propto \prod_{i}P\left(\mathrm{sampl}e_{i}|\Theta \right)\prod_{j}Q\left(\tau_{j}|\Theta \right)U\left(\varepsilon_{j}\right),$$

where Θ = {ε*_k_*, τ*_k_*}*_k_*_ = 1…_*_L_* and *U*(ε*_j_*) is our prior on the escape rates. We employ a Laplace prior *U*(ε) = exp(−Φε) parameterized by Φ favoring small escape rates. The prior regularizes the search for the minimum and results in conservative estimates of escape rates.

### Obtaining maximum likelihood estimates

Finding the set of escape rates and seed times that maximizes the posterior probability can be difficult due to multiple maxima and ridges in the high dimensional search space, and uncertainty remains. To ensure that the global optimum will be reliably discovered, we exploit the sequential nature of the dynamics and use the fact that earlier escapes strongly affect the timing of the later ones, but not vice versa. Thus adding genotypes with an increasing number of mutations one at a time results in a reasonable initial guess on top of which a global true multi-locus search can be performed.

**Figure 4:**
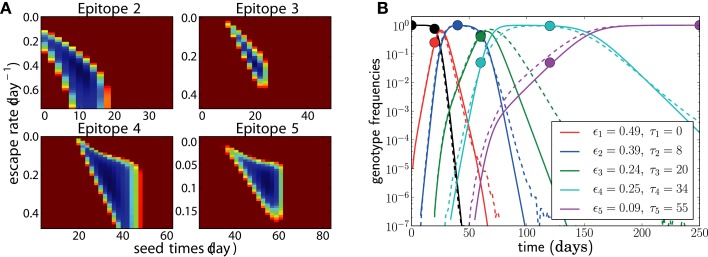
**Adding epitopes one by one is a feasible and reliable fitting strategy**. Assuming we know the population was homogeneous at *t* = 0, there is only one free parameter for the first epitope, which is easily determined. For all subsequent epitopes, we need to determine the seed time τ*_j_* and the escape rate ε*_j_*. In **(A)**, the negative log posterior probability of these parameters is shown for each of the epitopes. The surface typically exhibits a single minimum. **(B)** shows the genotype frequencies of the founder virus and the dominant escape variants (solid lines: model fit, dashed lines: actual simulated trajectories). The estimated escape rates of individual epitopes and the seed times of genotypes containing all escape mutations up to *j* are given in the legend. Only the samples indicated by balls (20 sequences at each time point) were used for the estimation. In this run, *N* = 10^7^, μ = 10^−5^, *r* = 0, and the escape rates ε*_j_* = 0.5, 0.4, 0.25, 0.15, 0.08 per day.

We have implemented such a search in Python, and the computationally expensive calculation of the posterior probability is implemented in C. The code infers parameters as follows:

- Fit the first escape assuming τ_1_ = 0 by a simple one dimensional minimization. This assumes that single mutants are already present in the population, consistent with the large viral population size present by the time a patient has been identified as HIV-1 infected (28, 29).
- Add additional epitopes successively by mapping the entire two-dimensional posterior distribution *P*(ε*_j_*, τ*_j_*) at fixed {ε*_k_*, τ*_k_*} for *k* < *j*. This step is illustrated in Figure 4A.
- Refine the estimates through local optimization via gradient descent, Monte Carlo methods, or local exhaustive search. The resulting parameters and trajectories are shown for one example in Figure 4B.
- Generate posterior distributions by Markov chain Monte Carlo (MCMC).

This procedure is described in more detail in the Section “Materials and Methods.” Fitting five epitopes takes on the order of a minute on one 2011 desktop machine (Apple iMac i7 2.93 GHz). Generating the local posterior distribution by MCMC takes roughly 20 min for 10^6^ steps.

### Comparison to simulated data

To evaluate the accuracy and reliability of our inference scheme, we performed true multi-locus stochastic simulations using FFPopSim (see Materials and Methods) and sampled genotypes from the simulation at a small number of time points. Time points and sample sizes were chosen to mimic patient data. We then inferred parameters from this “toy” data set and compared the result to the actual values. When interpreting these comparisons, it is important to distinguish two sources of error. First, limited sample size and sampling frequency will incur errors due to inaccurate estimates of the actual genotype frequencies from the sample. The second source of uncertainty is an inappropriate choice of model or model parameters. Such inappropriate model choices might include wrong estimates of the population size or mutation rates, the presence or absence of recombination, or time variable CTL activity.

We generate data assuming escape rates ε*_j_* = 0.5, 0.4, 0.25, 0.15, 0.08 per day and sample the population on days *t_i_* = 0, 20, 40, 60, 120, 250. An example of such samples is shown in Figure 2. Note that each genotype is typically only sampled at a single data point; it easily happens that a genotype is hardly seen at all. We therefore expect all inferences to be quite noisy as is the case with patient data.

#### Sample size and sampling frequency dependence

With more frequent and deeper sampling, inferring the model parameters is expected to become simpler. Indeed, as soon as each genotype is sampled more than once at intermediate frequency, one can estimate its growth advantage simply from its rate of increase. This is the rationale behind previous studies such as (4, 6). In many data sets, however, this condition is not met. By constraining the seed time based on the evolutionary trajectory of the previous escape, our method is able to produce a more accurate reconstruction of parameters with less data.

**Figure 5:**
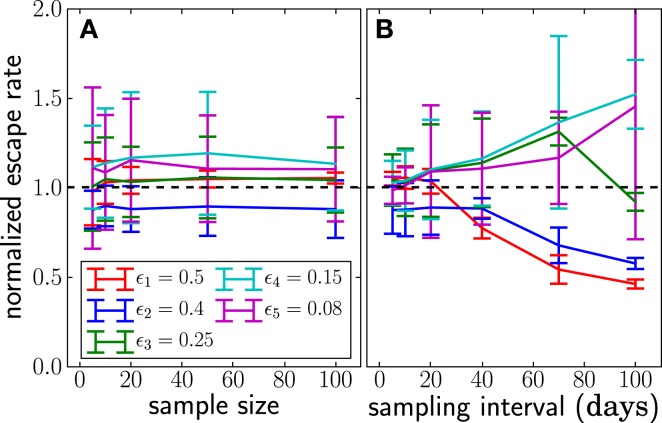
**The dependence of the accuracy of inference on sample sizes (A) and sampling intervals (B)**. The actual normalized escape rate is 1.0 and is shown by the dashed line. Sample size only moderately affects the accuracy, while sparse sampling (every 40 days in this example) leads to serious loss of accuracy. Sample size is *n* = 20 when sample intervals are varied, and sampling times are as illustrated in Figure 2 when sample size is varied. The plots show the mean ± one standard deviation. The actual values of the escape rates simulated are shown in the legend (same on both panels). In each run, *N* = 10^7^, μ = 10^−5^, and *r* = 0. Mean and standard deviation at each point are calculated from 100 independent simulations.

Figure 5 shows the estimates obtained as a function of the sampling frequency and sample size. Increasing the sample size improves the estimates only moderately, whereas increasing the sampling frequency leads to substantial improvements.

#### Model deviations

**Figure 6:**
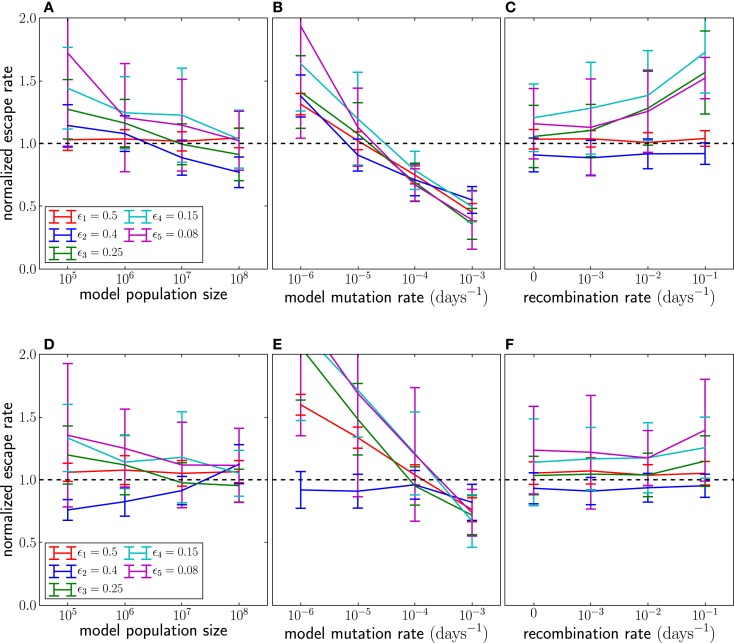
**The effect of assuming the wrong population parameters on the escape rate estimates**. To quantify the robustness against wrong assumptions, we simulate escape dynamics with parameters different from those assumed in the escape rate estimation. **(A–C)** show simulations with *N* = 10^7^ and μ = 10^−5^ per day, while **(D–F)** use a 10-fold higher mutation rate μ = 10^−4^. In **(C,F)**, the simulated recombination rate varies as shown. **(A,D)** Assuming a too small population size results in estimates that are too large. The effect is more pronounced at lower mutations rates. **(B)** Similarly, if the mutation rate is assumed too large, the estimated seeding of multiple mutants occurs too early and the estimates of escape rates are too low. Note that assuming the correct rates [μ = 10^−5^ in **(B)** and μ = 10^−4^ in **(F)**] results in unbiased estimates. **(C,F)** If the population recombines, the actual seed times are smaller than those estimated by the fitting routine. To compensate for the shorter time interval during which the escape variant rises, the estimates of escape rates are larger than the actual escape rates, at least at low mutation rates. For high mutation rates, recombination is less important because additional mutations are more efficient at producing multiple mutants than recombination. Mean and standard deviation at each point are calculated from 100 independent simulations.

The population size and the mutation rate explicitly enter our model through the seed time prior, but we rarely know these numbers accurately. Hence we need to understand how inaccurate assumptions affect our estimates. If we assume that *N*μ is larger than it really is, our inference method will favor seeding subsequent genotypes too early, which in turn results in erroneously small estimates of escape rates. We varied *N* and μ and observed the expected effect on the estimates as shown in Figure 6. The dependence on μ is stronger than that on *N*, since the effect of a larger population size is partly canceled by the longer time necessary to amplify the novel mutation to macroscopic numbers. However, even the dependence on μ is rather weak, and changing μ 10-fold only changes estimates of escape rates by ±50%. The underlying reason is that the seed times depend primarily on the logarithm of *N*μ⋅*Q*(τ*_j_*|γ*_j_*_-1_(*t*)) (see equation (4)), which peaks when *N*μγ*_j_*_ − 1_(*t*) ≈ 1. Because γ*_j_*_-1_(*t*) is growing exponentially, the position of the peak changes only logarithmically with the prefactor *N*μ. Changes in μ also affect the dynamics through the initial rise in frequency of novel genotypes due to recurrent mutations; see equation (3).

Another factor that affects seed times is recombination. HIV recombines via template switching following the coinfection of one target cell by several virus particles (21). In chronic infection, coinfection occurs with a frequency of about 1% (13, 14). Recombination is not modeled in the seed time prior of our inference method but can speed up escape by combining escape mutations at different epitopes. As a result, if recombination is present, seeding tends to happen earlier than our prior would suggest. If the model assumes that seeding occurs later than in reality, there is less time for an escape variant to grow to its observed frequency. Hence the estimated escape rate (growth rate) is larger than the actual escape rate to compensate for the shorter time. In Figure 6, we compare the estimates obtained by applying our inference method to simulation data with recombination. Recombination starts to have substantial effects once coinfection exceeds a few percent. Recombination primarily affects the incorporation of more weakly selected mutations and can be ignored for very strongly selected CTL escape mutations. Recombination also has negligible effects if the mutation rate is large as is seen in Figure 6F.

#### Unobserved intermediates and compensatory mutations

**Figure 7:**
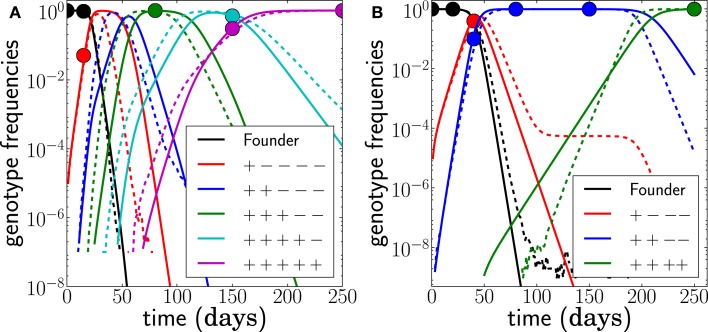
**Unobserved intermediates and compensatory mutations**. **(A)** shows a scenario where the genotype with only 2 escape mutations (blue) was not observed even though this genotype was transiently at high frequencies. We fit this scenario by assuming both mutations have the same escape rate but occur sequentially (*N* = 10^7^, *r* = 0, μ = 10^−5^). **(B)** shows a scenario where escape mutations 3 and 4 only occur together and any genotype containing only one of the two mutations is not viable. Hence the effective mutation rate into the genotype is μ^2^ = 10^−10^ and the waiting time for this genotype is longer. Note that the population size is *N* = 10^9^ in this example (*r* = 0, μ = 10^−5^). The last escape only appears once the previous escape mutations have reached frequency one, and the seeding time is quite variable.

The time intervals between successive samples are sometimes too large to observe the accumulation of single mutations, so the dominant genotype at one time point differs by more than one mutation from the previous. This can arise for two reasons. First, one or several unobserved genotypes may have transiently been at high frequency but been out-competed by later genotypes before the next sample was taken. Second, one escape might have required more than one mutation, for example because single mutants are not viable and a compensatory mutation is needed (30). Both scenarios can be accounted for in our scheme and are illustrated in Figure 7.

Unobserved, but individually beneficial, intermediate genotypes can be included by assuming they all have the same escape rate and were seeded one from the other. There is not sufficient information to estimate more than an average escape rate for all of them. For a given set of sampled frequencies, the estimated escape rates increase as more and more intermediates are assumed. Such unobserved intermediates are common in the data from infected individuals analyzed below.

Compensatory mutations and “multiple-hit” escapes can be accounted for by replacing the single site mutation rate in equation (4) by the effective rate at which the viable escape mutant appears. In the simplest case where all intermediate states are lethal and mutations are independent, this rate is simply the probability μ^*k*^, where *k* is the number of mutations needed. In other cases, the rates to multiple hits can be calculated using branching process approximations (31, 32). The choice of the relevant effective mutation rate for complex escapes must be made on a case-by-case basis. The effective mutation rate of a multiple-hit escape will often be low enough that its seed time is not very well constrained. If, for example, the population size is *N* = 10^8^ and the effective mutation rate is 10^−10^, the seed time distribution has a width of more than 100 days. Given this weak constraint, more data are required in order to estimate the escape rate accurately; see Figure 7.

### Immune escape in HIV-infected patients

Cytotoxic T-lymphocyte escape was characterized in detail in the patients CH58, CH40, and CH77 (7, 8) and further analyzed in Ganusov et al. (4). Sequences were obtained by single genome amplification followed by traditional sequencing. The data are sparser and less densely sampled than most of the artificial examples analyzed above, so any estimates are necessarily rather imprecise. Furthermore, we do not know exactly when infection occurred or CTL selection started. The days given in the above papers are relative to the date of identification of the patient as HIV infected. It has been estimated that in a chronically infected patient, there are a total of around 4 × 10^7^ infected cells (33). Hence, the population size is *N* ≈ 10^7^ but might be larger during peak viremia or smaller due to bottleneck effects or the myriad of factors influencing patient-to-patient variation in viral load. We determined posterior distributions for population sizes ranging from *N* = 10^5^ to *N* = 10^8^. The mutation rate was set to 10^−5^ per day (10). This value is appropriate if only one escape mutation per epitope is available. If escape can happen in many different ways, a higher rate of about μ = 10^−4^ per day should be used, so we repeated the estimation with μ = 10^−4^ finding similar results; see below. Both of these scenarios are observed (18). Recombination in HIV occurs but is not modeled here because its rate is low (13, 14), and it is expected to be less relevant for the strong escapes in large populations. In large populations, recurrent mutation is often more effective at accumulating escape mutations than recombination between two rare variants. Nevertheless, the neglect of recombination can lead to overestimation of escape rates; see above. Lastly, we assume that infection occurred τ = 20 days before the patient was identified and the viral population sampled (7).

For each patient, we initially considered all non-synonymous mutations that are eventually sampled at high frequency as potential candidates for sequential escape mutants. Nearby mutations in the same epitope were combined into one escape. We refined this list of candidates by considering only time points early in infection that were sampled with more than 5 genomes per time point and only the earliest 3-6 strong escapes. All samples used had between 7 and 15 sequences. The frequencies of these escape mutations and their linkage into multi-locus genotypes in the 5′ and 3′ half of the genome, which were sequenced independently, can be easily determined from the alignment provided in Salazar-Gonzales et al. (8). Linkage information between the 5′ and 3′ half genomes is missing but can in all cases be imputed using the assumption of sequential escapes. We ignored mutations whose frequency does not increase monotonically such as pol80 in subject CH40. Later in infection, there is extensive non-synonymous diversity and it is not feasible to fit a time course for most of these mutations.

In CH40 we considered samples at time points *t* = 0, 16, 45, 111, and 181 days and identified escape in six epitopes; the first escape occurs in nef185, followed by three indistinguishable escapes at gag113, gag389, and vpr74 and two additional escapes in vif161 and env145. Following Ganusov et al. (4) the number in the epitope name refers to the beginning of the 18-mer peptide covering the epitope. The mutation at env145 was not analyzed in Ganusov et al. (4), and 145 is simply the number of the mutated amino acid in gp120. The indistinguishable escapes gag113, gag389, and vpr74 are treated as described in the section on unobserved intermediates (all three escapes are assumed to have identical escape rates and only their seed times are varied). Note that the fifth escape at epitope vif161 shows almost the same escape pattern as the three indistinguishable escapes preceding it. The escape rates of gag113, gag389, vpr74, and vif161 should therefore be interpreted with care. In CH58 we considered samples at time points *t* = 0, 9, 45, and 85 days and identified four escapes; the first escape is at env581 and the second at env830, followed by nef105 and gag236. In CH77 we considered samples at time points *t* = 0, 14, and 32 days and identified four escapes, namely the first escape in tat55 and subsequent escapes in env350, nef17, and nef73.

**Figure 8:**
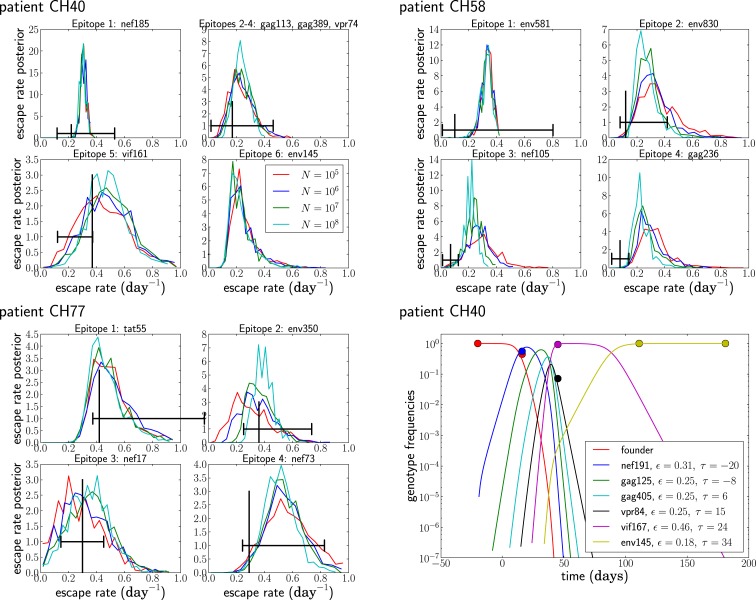
**The posterior distribution of the escape rates for different population sizes**. It is assumed that CTL selection starts 20 days prior to the date of identification, and the fitness prior has weight Φ = 10. The black vertical and horizontal lines indicate the estimates and confidence intervals obtained in Ganusov et al. (4). Note that the mutation env145 in CH40 was not analyzed in Ganusov et al. (4). The lower right panel shows the most likely genotype trajectories for patient CH40 with parameters *N* = 10^7^ and μ = 10^−5^. Each curve is labeled by an epitope but should be understood as the frequency of the genotype that has escaped at this and all previous epitopes. Note that no data are available to differentiate epitopes gag113, gag389, and vpr74. For those, we assume an arbitrary order and equal escape rates as explained in the section on unobserved intermediates.

Given the above assumptions, we obtained estimates for the seed time and escape rate of each mutation. For each patient, we obtained initial estimates using a naïve single epitope fit for each mutation; then, we iterated our multi-epitope fitting model five times. Next, we obtained posterior distributions for the escape rates, all shown in Figure 8, by performing a MCMC simulation using the likelihood function given in equation (5). After obtaining our estimates, we randomly changed the escape rates in increments of ±0.01 and the seed times by ±1, reevaluated the likelihood, and accepted the change with probability min(1, exp(Δ)), where Δ is the change in log-likelihood. The resulting Markov chain was run for 10^6^ steps with samples taken every 1000 steps.

Figure 8 shows the posterior distributions of the escape rates for different epitopes in the three patients evaluated assuming a mutation rate μ = 10^−5^ per day. Larger population sizes result in smaller estimates of the escape rates, as expected from Figure 6A. The posterior distributions for the first escapes are often very tight, but they depend on the time of the onset of CTL selection, which we have set here to *T* = 20 days prior to the first sample. If we assume that the time of the onset of CTL selection coincided with the first sample (i.e., *T* = 0), the estimates of escape rates of the first epitope ε_1_ are around 0.9, while later escapes are almost not sensitive to the choice of *T*.

**Figure 9:**
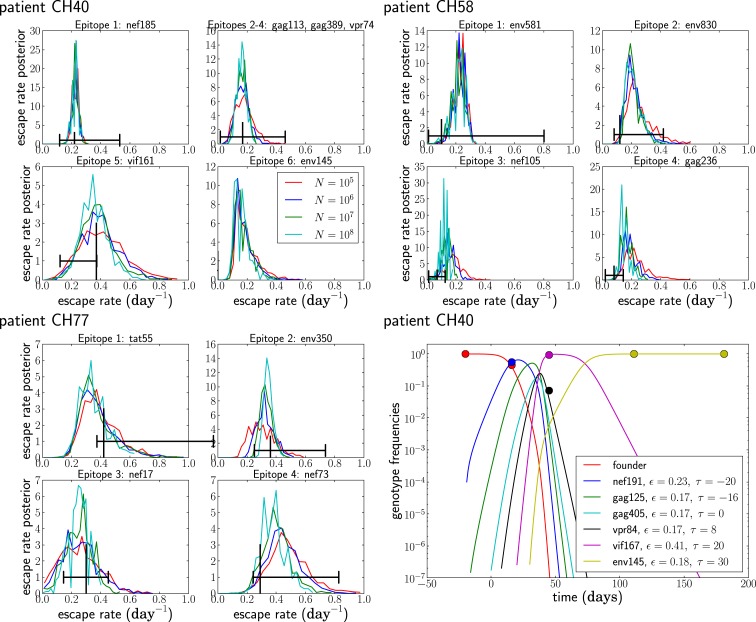
**Posterior distributions of escape rate assuming a mutation rate μ = 10^−4^ per day**. See Figure 8 for other details.

While the posterior distributions of escape rates of subsequent escape rates are quite broad, they nevertheless suggest that escape rates can be substantially higher than previously estimated (4, 6). Furthermore, the escape rate is not obviously negatively correlated with the time of emergence during acute infection with HIV-1, at least for the earliest four to six escapes. The underlying reason for this is that selection on a late escape is only active after the successful multiple mutant has been produced. In previous single epitope estimates, selection was allowed to act on the mutant frequency from the very beginning, resulting in a reduced estimate of the escape rate. Figure 8 also shows the inferred trajectories for the most likely parameter combination for patient CH40. One clearly sees the rapid rise and fall of multiple genotypes between the second and third time point. Given the large number of genotypes involved and the little data available, the escape rates estimated for this case are rather noisy. But this analysis clearly shows that strong selection is necessary to bring four mutations to fixation in just a few weeks. We repeated the analysis of the patient data assuming a mutation rate of μ = 10^−4^ and show the results in Figure 9. The overall picture is similar to what we found for μ = 10^−5^ per day, but escape rates tend to be lower.

## Discussion

We have suggested a way to infer viral escape rates from time series data sparsely sampled from the evolutionary dynamics of asexual or rarely sexual populations such as HIV. We exploit the sequential manner in which escape mutations accumulate, which allows us to constrain the times at which new escape mutations arose. These constraints regularize the inference to a large extent, but additional stability is gained by prioritizing small escape rates through an exponential prior.

Escape rates of single escape mutations have so far been estimated by comparing the time series data to a model that assumes logistic growth of the mutation with a constant rate. This approach has been used to analyze the intra-patient dynamics of recombinant HIV (34), drug resistance (35, 36), and CTL escape dynamics (4, 6, 20, 37, 38). While these methods work well if each mutation is sampled multiple times at intermediate frequencies, they provide very conservative lower bounds when data are sparse. Furthermore, they ignore the effects of competition between escapes at different epitopes and assume that each epitope can be treated independently. Since the recombination frequency in HIV is low (13, 14, 39), this can be a poor approximation. Our method improves on previous methods on both of these counts. We explicitly model the competition between escape mutations. This competition places constraints on the times at which genotypes with multiple escapes first arise (double mutants arise only after the single mutants), which makes the inference more robust and the lower bound tighter.

A related method to estimate CTL escape rates has been proposed by Leviyang (12), who modeled multiple escape mutations by an escape graph that is traversed by the viral population. Combining these two approaches, intra-epitope competition as modeled in (12) and the between epitope competition studied here, would be an interesting extension. Similar ideas have been developed in the context of mutations in cancer or evolution experiments (40).

While previous methods neglect interactions between epitopes altogether – equivalent to assuming very rapid recombination – our method ignores recombination during the inference. By comparison with simulations that include recombination, we have shown that neglecting recombination can result in overestimation of the escape rates by roughly 30% at plausible recombination rates of 1% (13, 14). We also show that neglecting recombination is less of a problem at higher mutation rates. Note that neglecting recombination cannot explain the larger escape estimates compared to previous studies. For patient CH58 we find escape rates that are up to threefold higher than earlier estimates (4), while we never see such a large deviation in our sensitivity analysis. Furthermore, the errors made when neglecting recombination for rapid early escapes are comparable to the uncertainties that result from infrequent sampling or more severe deviations of the model from reality, such as time variable CTL activity.

Reanalysis of CTL escape data from HIV using our method suggests that CTL escapes are substantially more rapid than previously thought. Even with a large prior against high escape rates (Φ = 10), we estimate that the escape rates of the first 4–6 escapes are on the order of 0.3-0.4 per day. The estimates at large population sizes are fairly insensitive to the prior for population sizes of 10^6^ or larger. Early in infection, it is plausible to assume that the relevant size is *N* = 10^7^ (28, 29, 41). If population sizes are small, relaxing the prior against high escape rates results in larger estimates, which further supports our finding that escape rates are often large and competition between escapes needs to be modeled. Given the sparse data, we can only estimate parameters of simple models and have to neglect many complicating features of HIV biology. Among other factors, the rate at which escape mutations are selected depends on the overall *R*_0_ of the infection, and CTL selection is probably time variable (4). The estimated parameters therefore represent time averaged effective escape rates.

The timing of escape has been shown to depend on epitope entropy and immunodominance (42). However, we modeled only the first four to six escapes in each patient, from which rather little information about differential timing can be obtained. In the case of CH77, the first four escapes occurred within a month from the identification of the patient. In patient CH58, it took roughly 3 months for four escapes to spread and the estimated escape rates are lower as expected. In the case of CH40, four of the six escapes show almost or completely indistinguishable escape patterns and we have little power to differentiate the escape rates at epitopes gag113, gag389, vpr74, and vif161. Hence any meaningful correlation with immunological features and epitope sequence conservation, i.e., low entropy, requires more data.

The proposed method to analyze multi-locus time series of adaptive evolution could be useful in many context where the genotypic compositions of large populations of viruses or cells can be monitored over time. Whenever mutations occur rapidly enough that they compete which each other, this competition has to be accounted for in the analysis. Outside of virus evolution, possible applications include the development of cancer and microbial evolution experiments.

## Materials and Methods

### Data preparation

Our fitting method uses counts *k_ij_* of genotypes *g_j_* at time points *t_i_* to infer escape rates of individual mutations. The procedure used to obtain successive genotype counts from sequence data sampled from patients is outlined in the text. As input data, our analysis scripts expect a white-space delimited text file with a format shown in Table 1. In addition, a separate file with the total number of sequences at each time point can be provided. This file is expected to have the same format as the matrix with the genotype counts; see Table 1. In absence of such a file, the sample sizes at each time point are obtained by summing the genotype counts.

**Table 1:** **Format of input data: the escape mutations are ordered first by the time of first observation and then by abundance**.

Time (days)	Founder	Env581	Env830	Nef105	Gag236
9	5	2	0	0	0
45	0	0	5	3	0
85	0	0	0	0	8

*Each entry in the table in a particular column reports the number of times a sequence is observed containing the escape of that column and all previous escape mutations*.

To test our method, artificial data *k_ij_* = *k*(*g_j_, t_i_*) were obtained from simulated trajectories (generated by FFPopSim) by binomial sampling (with size *n_i_*) at specified time points *t_i_*. Trajectory generation and sampling are implemented in the file model_fit/ctlutils.py at http://git.tuebingen.mpg.de/ctlfit; see below.

#### Sequence data

The HIV sequences for patients CH40, CH58, and CH77 where downloaded from http://www.hiv.lanl.gov/content/sequence/HIV/USER_ALIGNMENTS/Salazar.html (8).

### Inference

The inference procedure consists of *initial guessing*, *sequential addition of escapes*, *multi-dimensional refinement*, and estimation of *posterior distributions*. The implementation can be found in src/ctl_fit.py, with the C code for the likelihood calculation in src/cfit.cpp.

#### Initial guesses

We produce initial guesses by single epitope modeling. The frequency of each escape mutation, ν*_j_*, grows logistically with the escape rate (11). We expect that only the frequency of the first escape mutation is significantly affected by mutational input, because it receives input from the abundant founder sequence, whereas the later escapes only receive mutational input from the previously escaped genotype, which is still rare when the novel escape arises. Hence we only model the mutational dynamics of the first escape. In a single epitope model, the frequency of the founder variant is one minus the frequency of the escape variant. The frequency of the escape variant increases by μ(1 − ν_1_) per day due to mutations from the founder and decreases by μν_1_ due to further mutations to additional escapes. Combined with the logistic growth, the dynamics of ν_1_ is described by

(6) $${\dot{\nu }}_{1}\left(t\right)=\varepsilon_{1}\nu_{1}\left(1-\nu_{1}\right)+\mu \left[1-2\nu_{1}\right].$$

with initial condition ν_1_(0) = 0. Note the difference between the allele frequency ν, which refers to a particular escape mutation, and γ, which corresponds to frequencies of particular multi-epitope genotypes. The above ODE has the solution

(7) $$\nu_{1}\left(t\right)=\frac{1}{2\varepsilon_{1}}\left[\varepsilon_{1}-2\mu +R\tanh\left(\frac{\alpha +t}{2}R\right)\right]$$

where $R=\sqrt{\varepsilon_{1}^{2}+4\mu^{2}}$ and $\alpha =\frac{4\mu -2\varepsilon_{1}}{4\mu^{2}+\varepsilon_{1}^{2}}$ (11). The escape rate ε_1_ is determined by maximizing the likelihood (equation (5)) using fmin from scipy (43).

The seed time τ*_j_* of subsequent escape mutants *g_j_* is determined by maximizing the seed time prior *Q*(τ*_j_*|γ*_j_*_ − 1_) defined in equation (4) using the previously determined γ*_j_*_ − 1_. The frequencies of mutations are assumed to follow a logistic trajectory since the genotype from which they receive mutational input is itself still at low frequency:

(8) $$\nu_{j}\left(t\right)=\frac{e^{\varepsilon_{j}\left(t-\tau_{j}\right)}}{e^{\varepsilon_{j}\left(t-\tau_{j}\right)}+N\varepsilon_{j}}\;j>1.$$

Again, we maximize the posterior probability, equation (5), to obtain an initial estimate of ε*_j_*.

#### Sequential addition of escapes

Given the initial estimates for the first escape, we now add subsequent escapes to the multi-epitope model, which is formulated in terms of genotype counts *k_ij_* and frequencies γ*_j_*(*t*). Note that the interpretation of genotype counts depends on how many epitopes are modeled. For example, if we model epitopes 1, …, *j* out of a total of *L* epitopes, counts for genotype *j* are $k_{\mathrm{ij}}=\sum_{l=j}^{L}k_{\mathrm{il}},$ i.e., we ignore all later escapes.

If the added escape is unique, i.e., no other escape mutation has the exact same temporal pattern, we calculate the likelihood on a 21 × 31 grid of escape rates and seed times; comp. Figure 4. The grid spans values between 0 and twice the initial estimate for both the seed time and the escape rate. The most likely combination of seed time and escape rate is chosen, and the procedure is repeated with the next epitope.

If multiple epitopes exhibit the same temporal pattern, we add them all at once, constrain their escape rates to be equal, and assume they emerged in the order listed in the genotype matrix. Since we now have to optimize one joint escape rate and multiple seed times, we do not map the likelihood surface exhaustively but rather perform a greedy search. We examine next-neighbor moves with steps δτ = ± 1 day and δε = ± 0.02 per day, moves which change all seed times by δτ, and 20 moves in which all seed times and escape rates are changed by δτ and δε with random sign; the step that maximizes the likelihood is accepted. This is repeated until no favorable move is found and further repeated with δε = ±0.01 and ±0.001 per day.

#### Refinement

We then iterate sequentially over every epitope and optimize its seed time and escape rate as described above, but with all other epitopes part of the multi-epitope model. This typically leads to rather small adjustments and converges rapidly.

#### Posterior distributions

To determine the posterior distribution of the escape rates, we attempt to change all seed times and escape rates by δτ = ± 1 day and δε = ± 0.01 per day with random sign. The move is accepted with probability min(1, exp(Δ)), where Δ is the difference in log-likelihood before and after the change. We sample this Markov chain every 1000 moves and thereby map the posterior distribution of seed times and escape rates.

### Usage

All source code and scripts are available at http://git.tuebingen.mpg.de/ctlfit.

#### Building

The part of our method that is implemented in C and the python bindings can be built using make and the Makefile provided in the src directory. Prerequisites for building are python2.7, scipy, numpy, swig, and a gcc compiler.

#### Fitting

Given a text file with genotype counts specified as shown in Table 1, fitting is performed by calling the script fit_escapes.py with Python. Parameters can be set via command line arguments:

(9) pythonfit_escapes.py--inputdatafile

where --input specifies the file with the genotype counts. Other parameters can be modified in a similar manner. Running the script with the option --help prints a list of all parameters. The estimated escape rates and seed times as well as the sampled posterior distribution will be saved in the directory fit_escapes_output, unless otherwise specified.

## Conflict of Interest Statement

The authors declare that the research was conducted in the absence of any commercial or financial relationships that could be construed as a potential conflict of interest.

## Footnotes

^1^There is a brief period after the initial production of the mutation during which the dynamics is stochastic and the initial mutant establishes only with a probability roughly equal to ε*d*, where *d* = 2 days is the generation time (23). However, we find ε*d* ≈ 1 and ignore this complication.

## References

[1] McMichael AJ Borrow P Tomaras GD Goonetilleke N Haynes BF The immune response during acute HIV-1 infection: clues for vaccine development. Nat Rev Immunol (2009) 10(1):11 10.1038/nri2674 20010788 PMC3119211

[2] Fernandez CS Stratov I De Rose R Walsh K Dale CJ Smith MZ Rapid viral escape at an immunodominant simian-human immunodeficiency virus cytotoxic T-lymphocyte epitope exacts a dramatic fitness cost. J Virol (2005) 79(9):5721–31 10.1128/JVI.79.9.5721-5731.2005 15827187 PMC1082732

[3] Li B Gladden AD Altfeld M Kaldor JM Cooper DA Kelleher AD Rapid reversion of sequence polymorphisms dominates early human immunodeficiency virus type 1 evolution. J Virol (2007) 81(1):193–201 10.1128/JVI.01231-06 17065207 PMC1797245

[4] Ganusov VV Goonetilleke N Liu MKP Ferrari G Shaw GM McMichael AJ Fitness costs and diversity of the cytotoxic T lymphocyte (CTL) response determine the rate of CTL escape during acute and chronic phases of HIV infection. J Virol (2011) 85(20):10518–28 10.1128/JVI.00655-11 21835793 PMC3187476

[5] Seki S Matano T CTL escape and viral fitness in HIV/SIV infection. Front Microbiol (2012) 2:267 10.3389/fmicb.2011.00267 22319514 PMC3250645

[6] Asquith B Edwards CTT Lipsitch M McLean AR Inefficient cytotoxic T lymphocyte-mediated killing of HIV-1-infected cells in vivo. PLoS Biol (2006) 4(4):e90 10.1371/journal.pbio.0040090 16515366 PMC1395353

[7] Goonetilleke N Liu MKP Salazar-Gonzalez JF Ferrari G Giorgi E Ganusov VV The first T cell response to transmitted/founder virus contributes to the control of acute viremia in HIV-1 infection. J Exp Med (2009) 206(6):1253–72 10.1084/jem.20090365 19487423 PMC2715063

[8] Salazar-Gonzalez JF Salazar MG Keele BF Learn GH Giorgi EE Li H Genetic identity, biological phenotype, and evolutionary pathways of transmitted/founder viruses in acute and early HIV-1 infection. J Exp Med (2009) 206(6):1273–89 10.1084/jem.20090378 19487424 PMC2715054

[9] Perelson AS Neumann AU Markowitz M Leonard JM Ho DD HIV-1 dynamics in vivo: virion clearance rate, infected cell life-span, and viral generation time. Science (1996) 271(5255):1582–6 10.1126/science.271.5255.1582 8599114

[10] Mansky LM Temin HM Lower in vivo mutation rate of human immunodeficiency virus type 1 than that predicted from the fidelity of purified reverse transcriptase. J Virol (1995) 69(8):5087–94 7541846 10.1128/jvi.69.8.5087-5094.1995 PMC189326

[11] Ganusov VV Neher RA Perelson AS Mathematical modeling of escape of HIV from cytotoxic T lymphocyte responses. J Stat Mech (2013) 2013(1):P01010 10.1088/1742-5468/2013/01/P01010 PMC3961578 24660019

[12] Leviyang S Computational inference methods for selective sweeps arising in acute HIV infection. Genetics (2013) 194:737–52 10.1534/genetics.113.150862 23666940 PMC3697977

[13] Neher RA Leitner T Recombination rate and selection strength in HIV intra-patient evolution. PLoS Comput Biol (2010) 6(1):e1000660 10.1371/journal.pcbi.1000660 20126527 PMC2813257

[14] Batorsky R Kearney MF Palmer SE Maldarelli F Rouzine IM Coffin JM Estimate of effective recombination rate and average selection coefficient for HIV in chronic infection. Proc Natl Acad Sci U S A (2011) 108(14):5661–6 10.1073/pnas.1102036108 21436045 PMC3078368

[15] da Silva J The dynamics of HIV-1 adaptation in early infection. Genetics (2012) 190(3):1087–99 10.1534/genetics.111.136366 22209906 PMC3296244

[16] Keele BF Giorgi EE Salazar-Gonzalez JF Decker JM Pham KT Salazar MG Identification and characterization of transmitted and early founder virus envelopes in primary HIV-1 infection. Proc Natl Acad Sci U S A (2008) 105(21):7552–7 10.1073/pnas.0802203105 18490657 PMC2387184

[17] Fischer W Ganusov VV Giorgi EE Hraber PT Keele BF Leitner T Transmission of single HIV-1 genomes and dynamics of early immune escape revealed by ultra-deep sequencing. PLoS ONE (2010) 5(8):e12303 10.1371/journal.pone.0012303 20808830 PMC2924888

[18] Henn MR Boutwell CL Charlebois P Lennon NJ Power KA Macalalad AR Whole genome deep sequencing of HIV-1 reveals the impact of early minor variants upon immune recognition during acute infection. PLoS Pathog (2012) 8(3):e1002529 10.1371/journal.ppat.1002529 22412369 PMC3297584

[19] Petravic J Loh L Kent SJ Davenport MP CD4+ target cell availability determines the dynamics of immune escape and reversion in vivo. J Virol (2008) 82(8):4091–101 10.1128/JVI.02552-07 18272587 PMC2293002

[20] Ganusov VV De Boer RJ Estimating costs and benefits of CTL escape mutations in SIV/HIV infection. PLoS Comput Biol (2006) 2(3):e24 10.1371/journal.pcbi.0020024 16604188 PMC1420660

[21] Levy DN Aldrovandi GM Kutsch O Shaw GM Dynamics of HIV-1 recombination in its natural target cells. Proc Natl Acad Sci U S A (2004) 101(12):4204–9 10.1073/pnas.0306764101 15010526 PMC384719

[22] Zanini F Neher RA FFPopSim: an efficient forward simulation package for the evolution of large populations. Bioinformatics (2012) 28(24):3332–3 10.1093/bioinformatics/bts633 23097421 PMC3519462

[23] Markowitz M Louie M Hurley A Sun E Di Mascio M Perelson AS A novel antiviral intervention results in more accurate assessment of human immunodeficiency virus type 1 replication dynamics and T-cell decay in vivo. J Virol (2003) 77(8):5037–8 10.1128/JVI.77.8.5037-5038.2003 12663814 PMC152136

[24] Sunnker M Busetto AG Numminen E Corander J Foll M Dessimoz C Approximate Bayesian computation. PLoS Comput Biol (2013) 9(1):e1002803 10.1371/journal.pcbi.1002803 23341757 PMC3547661

[25] Basu D On the elimination of nuisance parameters. J Am Stat Assoc (1977) 72(358):355–66 10.1080/01621459.1977.10481002

[26] Kepler TB Perelson AS Modeling and optimization of populations subject to time-dependent mutation. Proc Natl Acad Sci U S A (1995) 92(18):8219–23 10.1073/pnas.92.18.8219 7667271 PMC41128

[27] Desai MM Fisher DS Beneficial mutation selection balance and the effect of linkage on positive selection. Genetics (2007) 176(3):1759–98 10.1534/genetics.106.067678 17483432 PMC1931526

[28] Coffin JM HIV population dynamics in vivo: implications for genetic variation, pathogenesis, and therapy. Science (1995) 267(5197):483–9 10.1126/science.7824947 7824947

[29] Perelson AS Essunger P Ho DD Dynamics of HIV-1 and CD4+ lymphocytes in vivo. AIDS (1997) 11(Suppl A):S17–24 9451962

[30] Read EL Tovo-Dwyer AA Chakraborty AK Stochastic effects are important in intrahost HIV evolution even when viral loads are high. Proc Natl Acad Sci U S A (2012) 109(48):19727–32 10.1073/pnas.1206940109 23112156 PMC3511713

[31] Weissman DB Desai MM Fisher DS Feldman MW The rate at which asexual populations cross fitness valleys. Theor Popul Biol (2009) 75(4):286–300 10.1016/j.tpb.2009.02.006 19285994 PMC2992471

[32] Neher RA Shraiman BI Genetic draft and quasi-neutrality in large facultatively sexual populations. Genetics (2011) 188:975–96 10.1534/genetics.111.128876 21625002 PMC3176096

[33] Haase AT Henry K Zupancic M Sedgewick G Faust RA Melroe H Quantitative image analysis of HIV-1 infection in lymphoid tissue. Science (1996) 274(5289):985–9 10.1126/science.274.5289.985 8875941

[34] Liu S-L Mittler JE Nickle DC Mulvania TM Shriner D Rodrigo AG Selection for human immunodeficiency virus type 1 recombinants in a patient with rapid progression to AIDS. J Virol (2002) 76(21):10674–84 10.1128/JVI.76.21.10674-10684.2002 12368309 PMC136598

[35] Paredes R Sagar M Marconi VC Hoh R Martin JN Parkin NT In vivo fitness cost of the m184v mutation in multidrug-resistant human immunodeficiency virus type 1 in the absence of lamivudine. J Virol (2009) 83(4):2038–43 10.1128/JVI.02154-08 19019971 PMC2643770

[36] Bonhoeffer S Barbour AD De Boer RJ Procedures for reliable estimation of viral fitness from time-series data. Proc Biol Sci (2002) 269(1503):1887–93 10.1098/rspb.2002.2097 12350250 PMC1691111

[37] Asquith B McLean AR In vivo CD8+ T cell control of immunodeficiency virus infection in humans and macaques. Proc Natl Acad Sci U S A (2007) 104(15):6365–70 10.1073/pnas.0700666104 17404226 PMC1851058

[38] Petravic J Ribeiro RM Casimiro DR Mattapallil JJ Roederer M Shiver JW Estimating the impact of vaccination on acute simian-human immunodeficiency virus/simian immunodeficiency virus infections. J Virol (2008) 82(23):11589–98 10.1128/JVI.01596-08 18799584 PMC2583643

[39] Josefsson L King MS Makitalo B Brännström J Shao W Maldarelli F Majority of CD4+ T cells from peripheral blood of HIV-1-infected individuals contain only one HIV DNA molecule. Proc Natl Acad Sci U S A (2011) 108(27):11199–204 10.1073/pnas.1107729108 21690402 PMC3131354

[40] Illingworth CJR Mustonen V A method to infer positive selection from marker dynamics in an asexual population. Bioinformatics (2012) 28(6):831–7 10.1093/bioinformatics/btr722 22223745 PMC3307107

[41] Boltz VF Ambrose Z Kearney MF Shao W Ramani VNK Maldarelli F Ultrasensitive allele-specific PCR reveals rare preexisting drug-resistant variants and a large replicating virus population in macaques infected with a simian immunodeficiency virus containing human immunodeficiency virus reverse transcriptase. J Virol (2012) 86(23):12525–30 10.1128/JVI.01963-12 22933296 PMC3497681

[42] Liu MKP Hawkins N Ritchie AJ Ganusov VV Whale V Brackenridge S Vertical T cell immunodominance and epitope entropy determine HIV-1 escape. J Clin Invest (2013) 123(1):380–93 10.1172/JCI65330 23221345 PMC3533301

[43] Oliphant T Python for scientific computing. Comput Sci Eng (2007) 9(3):10–20 10.1109/MCSE.2007.58

